# Increasing cellular fitness and product yields in *Pseudomonas putida* through an engineered phosphoketolase shunt

**DOI:** 10.1186/s12934-022-02015-9

**Published:** 2023-01-19

**Authors:** Lyon Bruinsma, Maria Martin-Pascual, Kesi Kurnia, Marieken Tack, Simon Hendriks, Richard van Kranenburg, Vitor A. P. Martins dos Santos

**Affiliations:** 1 Laboratory of Systems and Synthetic Biology, Wageningen University & Research, 6708 WE Wageningen, The Netherlands; 2 Corbion, 4206 AC Gorinchem, The Netherlands; 3 Laboratory of Microbiology, Wageningen University & Research, 6708 WE Wageningen, The Netherlands; 4 Laboratory Bioprocess Engineering, Wageningen University & Research, 6708 WE Wageningen, The Netherlands; 5 LifeGlimmer GmbH, 12163 Berlin, Germany; 6 Present Address: Faculty of Engineering and Natural Sciences, Tampere University, 33100 Tampere, Finland

## Abstract

**Background:**

*Pseudomonas putida* has received increasing interest as a cell factory due to its remarkable features such as fast growth, a versatile and robust metabolism, an extensive genetic toolbox and its high tolerance to oxidative stress and toxic compounds. This interest is driven by the need to improve microbial performance to a level that enables biologically possible processes to become economically feasible, thereby fostering the transition from an oil-based economy to a more sustainable bio-based one. To this end, one of the current strategies is to maximize the product-substrate yield of an aerobic biocatalyst such as *P. putida* during growth on glycolytic carbon sources, such as glycerol and xylose. We demonstrate that this can be achieved by implementing the phosphoketolase shunt, through which pyruvate decarboxylation is prevented, and thus carbon loss is minimized.

**Results:**

In this study, we introduced the phosphoketolase shunt in the metabolism of *P. putida* KT2440*.* To maximize the effect of this pathway, we first tested and selected a phosphoketolase (Xfpk) enzyme with high activity in *P. putida*. Results of the enzymatic assays revealed that the most efficient Xfpk was the one isolated from *Bifidobacterium breve*. Using this enzyme, we improved the *P. putida* growth rate on glycerol and xylose by 44 and 167%, respectively, as well as the biomass yield quantified by OD_600_ by 50 and 30%, respectively. Finally, we demonstrated the impact on product formation and achieved a 38.5% increase in mevalonate and a 25.9% increase in flaviolin yield from glycerol. A similar effect was observed on the mevalonate-xylose and flaviolin-xylose yields, which increased by 48.7 and 49.4%, respectively.

**Conclusions:**

*Pseudomonas putida* with the implemented Xfpk shunt grew faster, reached a higher final OD_600nm_ and provided better product-substrate yields than the wild type. By reducing the pyruvate decarboxylation flux, we significantly improved the performance of this important workhorse for industrial applications. This work encompasses the first steps towards full implementation of the non-oxidative glycolysis (NOG) or the glycolysis alternative high carbon yield cycle (GATCHYC), in which a substrate is converted into products without CO_2_ loss These enhanced properties of *P. putida* will be crucial for its subsequent use in a range of industrial processes.

**Supplementary Information:**

The online version contains supplementary material available at 10.1186/s12934-022-02015-9.

## Background

There is a pressing need to transition to a sustainable and biobased economy [31]. The deployment of micro-organisms to produce chemicals currently derived from fossil fuels is a promising green alternative to this end [28]. One of the key challenges when using micro-organisms as cell factories is the optimization of the titer, rate, and yield (TRY) to make the production process competitive with the petrochemical industry [22].

*Pseudomonas putida* is an attractive host that is increasingly being developed as a cell factory for a wide range of biotechnological applications [20, 29]. Its high metabolic versatility, ability to tolerate environmental stresses and wide use of a variety of carbon sources make this bacterium one of the laboratory workhorses that can fulfil the demands of industrial biotechnology [2, 25, 26].

In current industrial cultivation processes, feedstock costs remain one of the major bottlenecks [6]. Therefore, the substrate-to-product ratio must be as close to the theoretical maximum as possible. Many high-value products, such as isoprenoids, butanol, and polyketides, are derived from the intermediate acetyl–CoA, a key compound connecting the glycolysis and the tricarboxylic acid (TCA) cycle [10]. *P. putida*, like many other bacteria grown on glycolytic carbon sources, produces acetyl-CoA through pyruvate decarboxylation. However, in this process, carbon is lost in the form of CO_2_, lowering the theoretical carbon yield. Nature has cunning ways to resolve this problem by itself, through the usage of phosphoketolases (Xfpks). These enzymes are widely distributed among *Bifidobacteria*. These bacteria lack the aldolase and glucose-6-phosphate NADP^+^ oxidoreductase enzymes and use an alternative route to metabolize carbohydrates, in which phosphoketolases are key. These enzymes irreversibly cleave the sugar phosphates xylulose-5-phosphate (X5P) and fructose-6-phosphate (F6P) to glyceraldehyde-3-phosphate (G3P) and erythrose-4-phosphate (E4P) respectively, releasing an acetyl-phosphate (AcP) in the process. This AcP is then converted by the phosphotransacetylase (Pta) to acetyl-CoA, circumventing pyruvate carboxylation [17]. One of the most famous examples of using this enzyme is the non-oxidative glycolysis (NOG) [5, 19]. The NOG pathway generates acetyl-CoA from sugars or sugar phosphates without carbon loss. To such an end, the Xfpk catalyzes the two aforementioned reactions, in which E4P and G3P are formed. To achieve complete carbon conservation, three molecules of F6P are needed. These are broken down into three AcP and two E4P molecules. The E4P molecules need to undergo carbon rearrangement by the transaldolase and transketolase reactions to regenerate two molecules of F6P.

Recent metabolic engineering efforts have shown that the implementation of a phosphoketolase can improve carbon yields and the production of acetyl-CoA–derived products. For instance, the introduction of a phosphoketolase from *B. adolescentis* into *E. coli* showed improved yields for poly-β-hydroxybutyrate (PHB) (63.7%), fatty acid (14.36%) and mevalonate (64.3%) [33]. Overexpression of the phosphoketolase gene from *B. animalis* in a *C. glutamicum* strain resulted in a 14% increase in the glutamic acid yield from glucose as well as suppression of CO_2_ emission [8]. Similarly, Meadows et al. [21] combined a xylulose-5-phosphate-specific phosphoketolase and three other heterologous enzymes to rewire the central carbon metabolism for more acetyl-CoA supply in *S. cerevisiae*, resulting in engineered strains that produce more farnesene and require less oxygen.

In this study, we first characterized several Xfpk enzymes from *Bifidobacterium* species through in vitro assays. Secondly, we demonstrate that the introduction of the phosphoketolase shunt in *P. putida* KT2440 can improve biomass formation on glycerol and xylose, consuming less substrate in the process. At last, we prove that the carbon-conserving pathway can significantly increase the yield of the acetyl-CoA-derived compounds, such as malonyl-CoA and mevalonate.

## Results

### Selecting the best phosphoketolase candidate

**Fig. 1:**
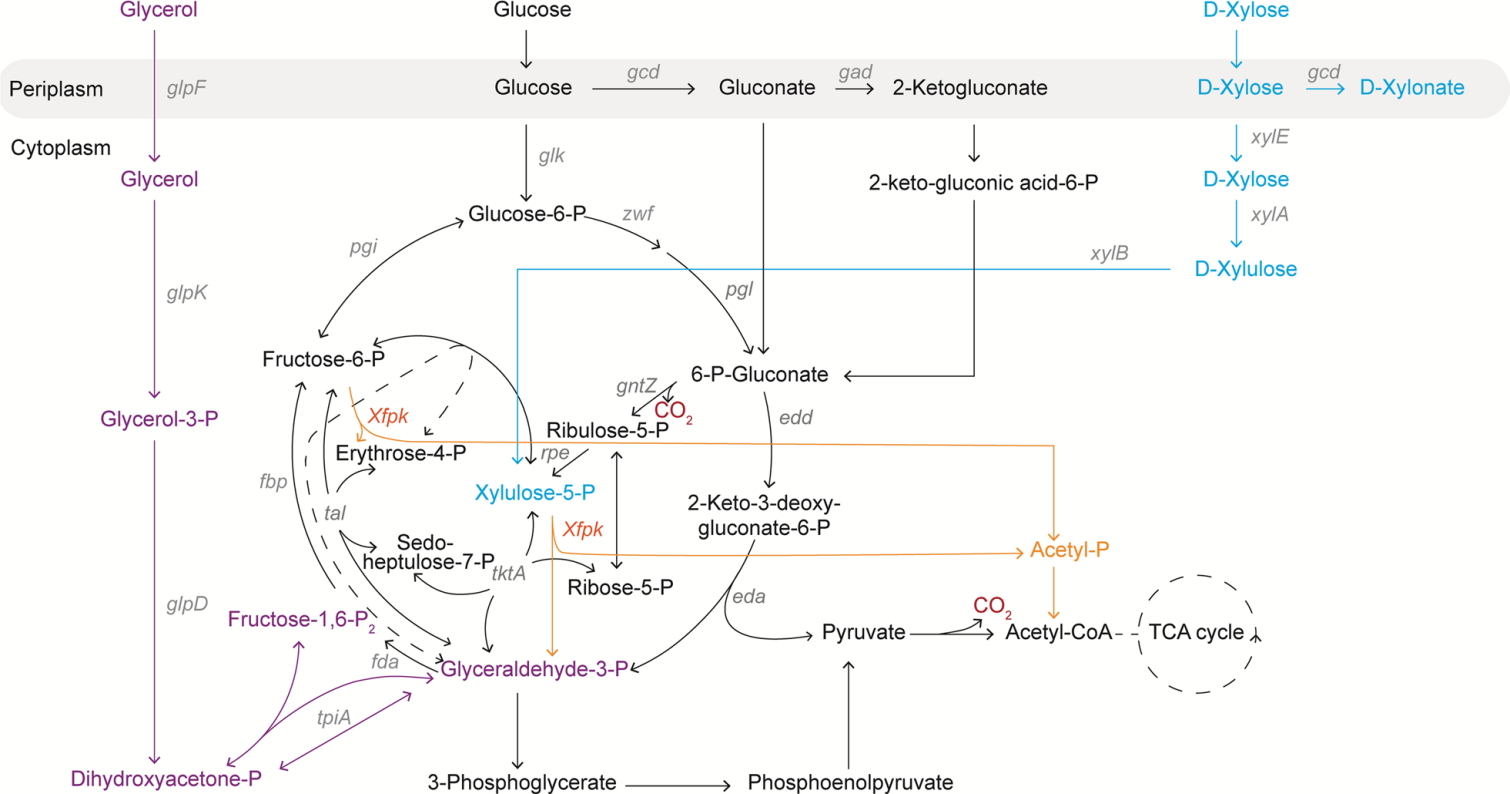
Metabolism of *P. putida* with the phosphoketolase shunt. Native metabolism of *P. putida* when glycerol (purple), glucose (black) and xylose (blue) are used as carbon sources. Rewired metabolism of *P. putida* when the phosphoketolase (Xfpk) is implemented (orange)

To maximize carbon conservation in *P. putida*, we introduced the phosphoketolase shunt in its central metabolism. Xfpk cleaves the respectisugar phosphates F6P and X5P and releases AcP in the process, which can be directly converted to acetyl-CoA. We hypothesized that through this introduction, pyruvate decarboxylation would be circumvented, and the carbon loss minimized (Fig. 1).

**Fig. 2:**
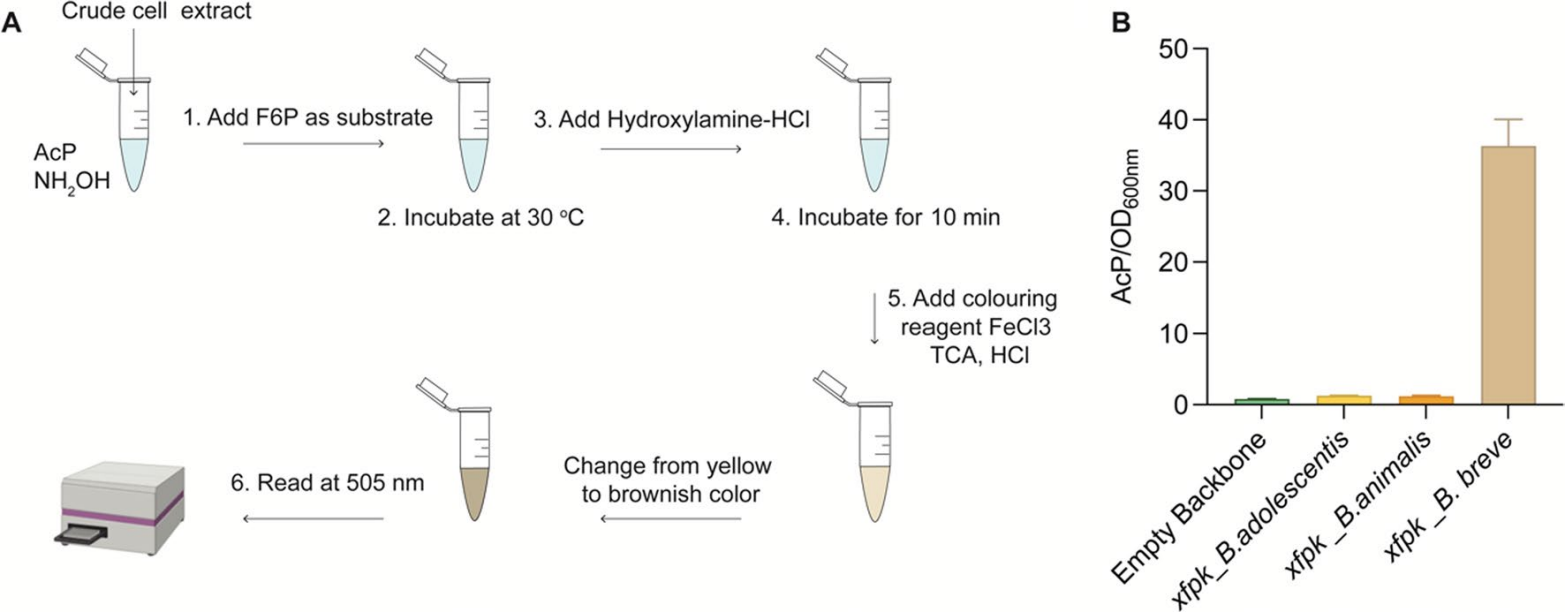
In vitro essay characterization of Xfpks. **A** Workflow of the ferric hydroxamate method used for Acp quantification. **B** Acp production from F6P in cell-free extracts by the three isolated Xfpk enzymes. Bar graphs represent the mean value ﻿ ± SD from three independent experiments

To select an Xfpk candidate with high enzymatic activity, an in vitro assay was performed. We cloned all candidate genes (*xfpk* from *B. adolescentis*, *B. animalis* and *B. breve*) under the control of a strong constitutive promoter (BBa_J23100) and RBS (0034) in the medium copy number pSEVAb83 vector. Functional expression of all enzymes in *P. putida* was assessed by the detection of AcP generated from F6P in cell-free extracts (Fig. 2). The Xfpk enzymes from *B. adolescentis* and *B. animalis* produced 1.26 and 1.19 mM AcP/OD_600_, respectively. Both only produced amounts slightly higher compared to the empty vector control (0.80 mM). This suggests that both enzymes are active, yet do not cleave F6P with high efficiency. However, the Xfpk from *B. breve* displayed high reactivity towards F6P. An AcP concentration of 36.25 mM/OD_600_ was measured, roughly 30-fold higher than the concentrations of the other two candidates. Therefore, the Xfpk from *B. breve* was selected for further experiments to assess growth and production.

### Effect of the phosphoketolase on the glycerol metabolism of *P putida* KT2440

**Fig. 3:**
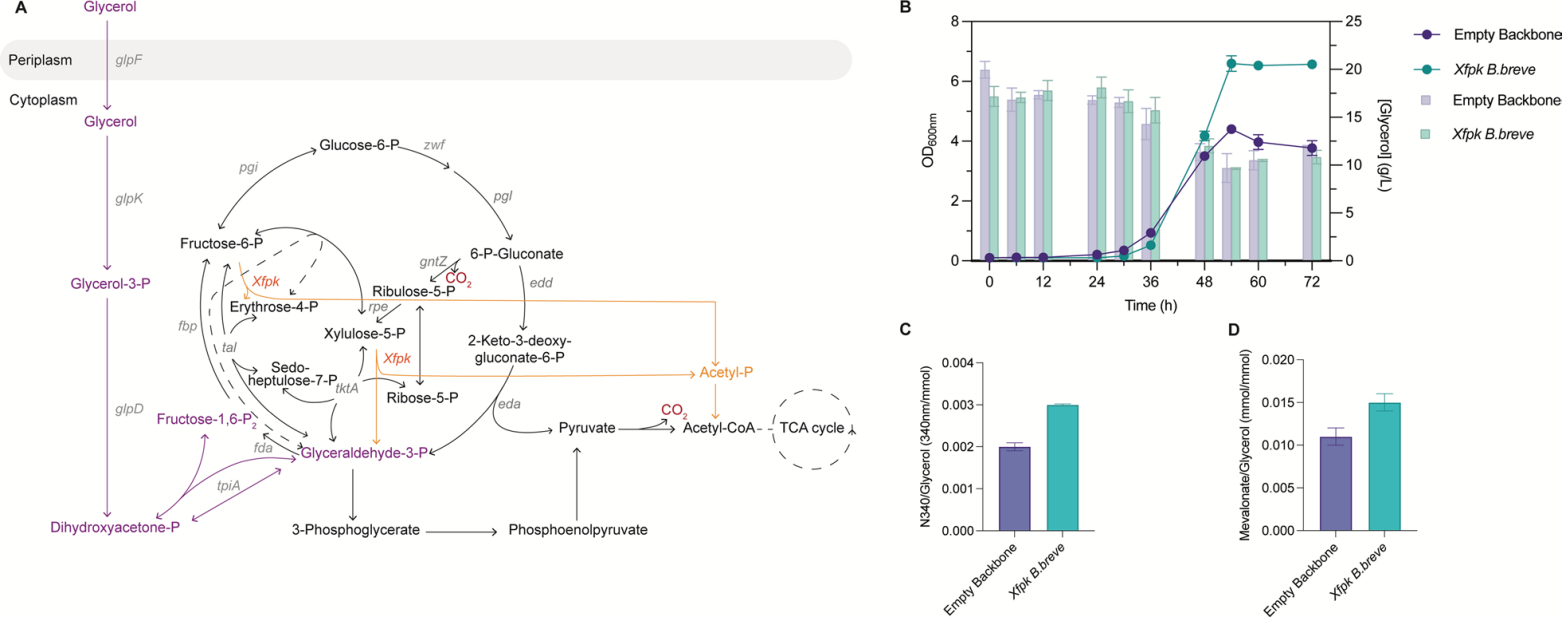
The effect of Xfpk expression on glycerol metabolism (**A**) Architecture of the metabolism of *P. putida* with the Xfpk when it is grown on glycerol, (**B**) growth patterns of strain growing on glycerol expressing an empty plasmid or the Xfpk. Lines present optical density and bars glycerol concentration, (**C**) Flaviolin yield, (**D**) Mevalonate yield. Data points and bar graphs represent the mean value ﻿ ± SD from three independent experiments

To determine whether the expression of the Xfpk from *B. breve* impacts the fitness of *P. putida*, we assessed its effect on cells grown on glycerol. Glycerol is a major waste product of the biodiesel industry and has a higher degree of reduction than glucose, making it an excellent substrate for microbial fermentation [25, 26]. To facilitate growth on glycerol, we constructed a *glpR* knockout mutant. This gene encodes a glycerol repressor and causes inconsistent lag phases when cultured on glycerol [23, 27]. Subsequently, *P. putida* Δ*glpR* strains, harbouring either an empty pSEVAb83 plasmid or the pSEVAb83 encoding the *xfpk* from *B. breve*, were grown in M9 minimal medium supplemented with 200 mM glycerol. The growth experiments revealed that Xfpk overexpression resulted in a 44.3% increase in specific growth rate from 0.12 to 0.18 h^−1^. Moreover, the introduction of the Xfpk increased the maximum OD_600nm_ from 4.4 to 6.6, an increase of 50% (Fig. 3B). We assumed that this increase could be an effect of enhanced glycerol consumption. However, both strains consumed glycerol to a similar extent, implying that most formed biomass is a direct result of carbon conservation (Fig. 3b). To use the phosphoketolase shunt to a further extent, we overexpressed the fructose-1,6-biphosphatase (Fbp) to increase the flux towards F6P. However, this did not have any added benefit over overexpressing the Xfpk alone (Data not shown).

### Exploiting the phosphoketolase shunt for the production of acetyl-CoA-derived compounds from glycerol

After demonstrating the beneficial effect of the Xfpk on growth, we shifted to exploit this advantageous strain to produce acetyl-CoA-derived compounds from glycerol. A wide variety of interesting valuable compounds can be derived from the intermediate acetyl-CoA e.g., isoprenoids, 1-butanol, and polyketides [10]. As described previously, in a normal glycolytic regime, acetyl-CoA is produced by pyruvate decarboxylation. In this conversion, carbon is lost in the form of CO_2_, negatively impacting the yield of acetyl-CoA-derived products. By rewiring the metabolic flux through the phosphoketolase shunt, this loss can be prevented, improving yields in the process. To assess this beneficial effect, we took two acetyl-CoA-derived compounds, malonyl-CoA, and mevalonate, as a proof of concept. Malonyl-CoA is a malonic acid that can be used to produce fatty alcohols and is derived directly from acetyl-CoA by the enzyme acetyl-CoA carboxylase consisting of four subunits (AccABCD).

For easy detection of increased malonyl-CoA levels, we used the previously repurposed type III polyketide synthase RppA [35]. This enzyme converts five molecules of malonyl-CoA to 1,3,6,8-tetrahydroxynapthaene, which is subsequently nonenzymatically oxidized to flaviolin. The produced flaviolin is red-coloured by itself, allowing spectrophotometrically quantification at a wavelength of 340 nm. Within our experiments, we overexpressed both *rppA* as well as all the several native subunits in *P. putida.*

The second acetyl-CoA-derived compound, mevalonate, is a key compound in industrial biochemistry. It is a metabolic precursor for terpenoids, which can be used in the production of cosmetics and biofuels [36]. Mevalonate is produced from acetyl-CoA in a three-step process. First one acetyl-CoA is converted to acetoacetyl-CoA. Secondly, a second acetyl-CoA will condense with acetoacetyl-CoA to form 3-hydroxy-3-methylglutaryl-CoA (HMG-CoA). Finally, this HMG-CoA is converted to mevalonate. For our experiments, we used the *mvaE* and *mvaS* genes from *Enterococcus faecalis.* The *mvaE* gene encodes a bifunctional protein that catalyzes both the first and last reaction in the mevalonate production pathway [36].

For the production experiments, we relocated the *xfpk* to a pSEVA62b vector and all product expression genes were cloned under a constitutive J23100 promoter into a pSEVA23b vector.

The production experiments for malonyl-CoA revealed a yield of 0.002 and 0.003 (absolute absorbance flaviolin/consumed glycerol) for *P. putida* Δ*glpR* with an empty plasmid and with the Xfpk shunt, respectively (Fig. 3C). Therefore, *P. putida* Δ*glpR* with the Xfpk shunt produced 38.5% more than the control. Similar results were obtained for mevalonate production, in which *P. putida* Δ*glpR* with an empty plasmid had a yield of 0.011 and with the Xfpk shunt of 0.015 mol/mol: 25.9% higher (Fig. 3D).

### Effect of the phosphoketolase on engineered xylose metabolism in *P putida* KT2440

The Xfpk is a promiscuous enzyme, which besides cleaving the sugar-phosphate F6P, also cleaves X5P. As X5P is the breakdown product of xylose degradation, we hypothesized that introducing the phosphoketolase shunt could enhance growth on xylose. (see Additional file 1) Xylose is a major constituent of hemicellulose which has been proposed as an alternative microbial feedstock [30]. Xylose utilization requires a combination of two genes, *xylA* encoding xylose isomerase and *xylB* encoding xylulokinase. Additional overexpression of *xylE* which encodes a xylose/H^+^ symporter has been described to improve the growth on xylose even further [12, 13]. The xylose utilization genes, derived from *E. coli*, were codon-optimized for *P. putida* using the Jcat tool (Additional file 2: Table S3). The *xylABE* genes alone and together with the *xfpk* were cloned in the low copy number vector pSEVAb62 under the expression of the strong constitutive BBa_J23100 promoter. Both plasmids were transformed in a *P. putida* strain with a Δ*gcd* background. The *gcd* gene encodes a glucose dehydrogenase, which has been reported to break down xylose to xylonate, a dead-end product [12].

**Fig. 4:**
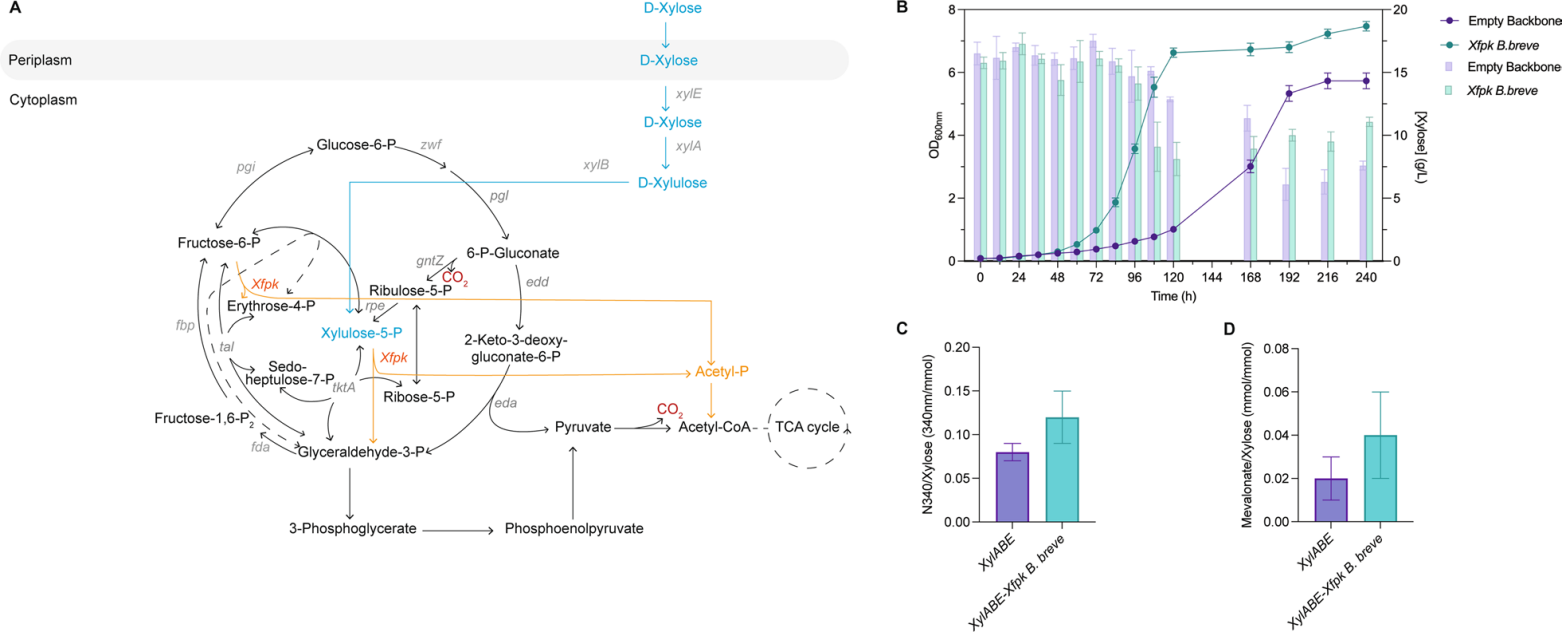
The effect of Xfpk expression on engineered xylose metabolism (**A**) Architecture of the metabolism of *P. putida* with the Xfpk when grown on xylose. **B** growth patterns of strains growing on xylose expressing an empty plasmid or the Xfpk. Lines present optical density and bars xylose concentration. **C** Flaviolin yield, (**D**) Mevalonate yield. Data points and bar graphs represent the mean value ﻿ ± SD from three independent experiments

Plasmid-born expression of the xylose utilization genes resulted in very long lag phases (> 312 h) and irregular growth patterns (Data not shown). Therefore, we decided to chromosomally express the operons. The xylose operons, both with and without the *xfpk*, and under the control of the constitutive Ptac promoter were chromosomally integrated into KT2440 Δ*gcd* downstream of the PP_5322 gene, resulting in strains KT2440Δ*gcd*-*xylABE* and KT2440Δ*gcd*-*xylABE*-*xfpk*. This locus has been described for its high basal expression, yet low impact on cellular fitness [7]. These new plasmid-free strains showed a reduced lag phase of 12 h. Corresponding with the results of the glycerol experiment, strains expressing the Xfpk, showed a faster growth rate and higher cell density compared to their non-expressing counterpart. KT2440Δ*gcd*-*xylABE* grew with a specific growth rate of 0.02 h^−1^ and reached the stationary phase after 216 h at an OD_600nm_ of 5.73. On the contrary, KT2440Δ*gcd*-*xylABE*-*xfpk* had a specific growth rate of 0.05 h^−1^ and a final OD_600nm_ of 7.4; an increase of 167% and 30.2%, respectively. Moreover, KT2440Δ*gcd*-*xylABE*-*xfpk* reached a higher OD while using less substrate than its non-expressing counterpart, highlighting the major impact carbon conservation has (Fig. 4B). As with glycerol, we equipped the xylose strains with the plasmids to produce malonyl and mevalonate. The production experiments for malonyl-CoA revealed a yield of 0.08 and 0.12 (absolute absorbance flaviolin/consumed xylose) for KT2440Δ*gcd*-*xylABE* and KT2440Δ*gcd*-*xylABE*-*xfpk*, respectively (Fig. 4C). Therefore, KT2440Δ*gcd*-*xylABE*-*xfpk* produced 49.4% more than the control. Similar results were obtained for mevalonate production, in which KT2440Δ*gcd*-*xylABE* had a yield of 0.022 and KT2440Δ*gcd*-*xylABE*-*xfpk* 0.042 mol/mol: 48.7% higher (Fig. 4D).

## Discussion

In this study, we showed that the introduction of the phosphoketolase shunt increased the cellular fitness of *P. putida*, enabling it to reach higher cell densities and growth rates. Additionally, yields were enhanced due to carbon conservation. Although we only showcase its beneficial effect on glycerol and xylose, we believe its catabolic repertoire can be further extended. Hemicellulose, of which xylose is a major constituent, contains other promising substrates that would profit from the phosphoketolase shunt, such as arabinose, mannose, and galactose [30]. For arabinose, Elmore et al. [13] recently engineered both the oxidative and isomerase pathway in *P. putida*. The oxidative pathway breaks down arabinose to the TCA intermediate 2-ketoglutarate, whilst the isomerase pathway is analogous to the xylose pathway. Therefore, for the Xfpk shunt to be used to its maximum efficiency, only the isomerase pathway, which has X5P as a product, would suffice. The other two substrates, mannose, and galactose, both break down to F6P, allowing full utilization of the Xfpk shunt as well.

The Xfpk shunt, as described in this study, could be further enhanced towards a full NOG or the recently engineered GATCHYC (glycolysis alternative high carbon yield cycle) [5, 16]. Both cycles display full carbon conservation with a 100% acetyl-CoA yield from its designated substrate. However, the NOG pathway contains two bidirectional steps (transaldolase and transketolase), which kinetically limits the pathway [1]. On the other hand, the GATCHYC relies mostly on unidirectional steps and the promiscuous activity of the Xfpk, which would cleave the sugar-phosphate sedoheptulose-7-phosphate (S7P) as well. The Xfpk from *B. breve* has been reported to use X5P and F6P in a 3:2 ratio [4]. It is likely that it also possesses a side activity towards S7P, making the implementation of the GATCHYC noteworthy. However, whereas full conversion of substrate to acetyl-CoA is a major benefit in both pathways, their major bottleneck is the insufficient formation of redox cofactors. To resolve this problem, alternative electron donors could potentially be added. One of the most promising candidates is formate, which can be directly derived from the electrochemical reduction of CO_2_ [9]. Moreover, it has already been showcased that supplementation of formate to *P. putida* KT2440 drastically increased NADH formation [37] Therefore, a combination of this carbon conservation pathway with formate might yield a superior platform strain.

Another recently engineered route to overcome the redox limitation of the NOG is the EP-bifido pathway [33]. This route was demonstrated with glucose as a carbon source and pushes the flux towards the oxidative pentose phosphate pathway. In this pathway, NADPH is generated at the expense of 1 CO_2_. However, due to the introduction of the Xfpk, more carbon loss was eventually prevented, and product yield was enhanced. The metabolism of *P. putida* consists of a cyclic architecture, termed EDEMP, merged from the Enter-Doudoroff (ED), the Embden-Meyerhof-Parnas (EMP) and the Pentose Phosphate (PP) pathway [23, 27]. This cycle recycles hexose phosphates, generating reducing equivalents in the form of NADPH. Replacing the EDEMP with the EP- bifido pathway could have great implications for the metabolism of *P. putida* as it would produce reducing equivalents whilst conserving carbon. This synergistic effect could further enhance product formation. Moreover, as NADPH is a well-known combater of oxidative stress, it could even further increase *P. putida* as an industrial workhorse [24].

## Conclusion

Native glycolytic routes are limited by low carbon efficiencies, largely due to carbon losses associated with pyruvate decarboxylation to acetyl-CoA. The introduction of the Xfpk shunt from *Bifidobacteria* offers a strategic bypass to redirect the main glycolytic flux to enhance carbon yields. In this study, we screened and assessed the activities of three Xfpk enzymes in *P. putida*. We showcased the exemplary effect carbon conservation can have on cellular fitness and production in *P. putida* when grown on glycerol or xylose. ·Altogether, this work provides a framework to further build upon this carbon-conserving mechanism to strengthen *P. putida* as an industriophile.

## Material & methods

### Bacterial strains and growth conditions

*P. putida* and *E. coli* cultures were incubated at 30 °C and 37 °C, respectively. For genetic modification and plasmid isolation, strains were cultured in Lysogeny broth (LB) medium containing 10 g/L NaCl, 10 g/L tryptone and 5 g/L yeast extract, unless otherwise indicated. For the preparation of solid media, 1.5% (*w*/*v*) agar was added. Antibiotics were used whenever required at the following concentrations: kanamycin (Km) 50 μg/ml, gentamycin (Gm) 10 μg/ml, chloramphenicol (Cm) 50 μg/ml and apramycin (Apra) 50 μg/ml. All growth and production experiments were performed using M9 minimal medium (per Liter; 3.88 g K_2_HPO_4_, 1.63 g NaH_2_PO_4_, 2.0 g (NH_4_)_2_SO_4_, pH 7.0) or MOPS media. The M9 media was supplemented with a trace elements solution (per Liter; 10 mg L^− 1^ ethylenediaminetetraacetic acid (EDTA), 0.1 g/L MgCl_2_.6H_2_O 2 mg/L ZnSO_4_7H_2_O, 1 mg/LCaCl_2_∙2H_2_O, 5 mg/LFeSO_4_∙7H_2_O, 0.2 mg/L, Na_2_MoO_4_∙2H_2_O, 0.2 mg/L CuSO_4_∙5H_2_O, 0.4 mg/L CoCl_2_∙6H_2_O, 1 mg/L MnCl_2_∙2H_2_O). In these experiments, strains were precultured in 10 ml LB with corresponding antibiotics. Then, the cultures were washed twice in M9 media or MOPS without a carbon source. Finally, the cultures were diluted to an OD_600_ of 0.1 to start the experiment and incubated in a rotary shaker at 200 rpm at 30 °C. Samples were taken at various time points for quantification of cell density and HPLC analysis.

### Plasmid construction

DNA fragments were amplified using Q5 Hot Start High Fidelity DNA Polymerase (New England Biolabs) and separated by electrophoresis using a 1% (*w*/*v*) agarose gel. DNA was purified by the NucleoSpin Gel and PCR clean-up kit (Macharey-Nagel, Germany). Plasmids were constructed using Golden Gate using the SEVA assembly protocol or through Gibson Assembly [11, 14]. The phosphoketolase genes were obtained from the genomic DNA of the *Bifidobacterium* strains: *B. animalis* DSM 10140*, B. adolescentis* ATCC 15703*, B. breve* DSM 21213 (Additional file 2, Table S3). The *rppA* gene from *Streptomyces Griseus* and all *accA-D* genes from *P. putida* KT2440 were taken from an in-house plasmid (Batianis et al., unpublished). The *mvaE* and *mvaS* genes were derived from pSMART-MEV1 ordered from Addgene (pSMART-Mev1 was a gift from Michael Lynch (Addgene plasmid #65815; http://n2t.net/addgene:65815; RRID: Addgene_65815)). All plasmids were transformed via heat shock in chemically competent *E. coli* Dh5α *λpyr* and transformants were selected on LB plates with corresponding antibiotics. Colonies were screened by colony PCR using Phire Hot Start II DNA Polymerase (Thermo Fisher Scientific Inc. Waltheim, MA, USA). The plasmids from successful screenings were extracted and verified by Sanger DNA sequencing (MACROGEN Inc, the Netherlands). Correct plasmids were transformed into *P. putida* using electroporation.

### Strain construction

The deletion and introduction of genes in the genome were performed using the protocol previously described by [34]. First, regions of approximately 500 bp upstream and downstream of the target genes were amplified from the genomic DNA of *P. putida* KT2440. The TS1 and TS2 fragments were cloned into the non-replicative pGNW plasmid and propagated into *E. coli* Dh5α *λpir.* Positive colonies were transformed into *P. putida* via electroporation and successful cointegrations were screened by PCR. For the chromosomal integration of the xylose operon, the TS1 and TS2 regions of the PP5322 locus were amplified. The xylose operon was assembled using the standard protocols of the previously described SevaBrick assembly [11]. Subsequently, this operon was cloned together with the TS1 and TS2 regions of the PP_5322 locus in the pGNW vector for µchromosomal integration. The second plasmid pQURE6-H [32] was introduced to the selected co-integrate. The transformants were grown on an LB agar plate with Gm and induced with 2 mM 3-methylbenzoic acid (3-mBz). The 3-*m*Bz was used to induce the XylS-dependent *Pm* promoter, driving the expression of the I-SceI homing nuclease. Deletions and insertions were confirmed by colony PCR. Successful recombinants were cured of pQURE6-H by omitting 3-mBz from the medium and selected for antibiotic sensitivity.

### Crude cell extraction

Cell-free extracts were obtained from 25 ml of culture broth in 50 ml Greiner Tubes and cultivated at 30 °C, 200 rpm orbital shaking until an OD600 of ca. 0.5–0.6. The cultures were pelleted by centrifugation for 10 min at 4 ºC. Cell pellets were washed once with 50 ml of precooled 1X PBS buffer (pH = 7.0) with 10 mM 2-mercaptoethanol at 4 ºC. The pellets were resuspended in 750 L of pre-cooled 1X PBS buffer (pH = 7.0) with 10 mM 2-mercaptoethanol. After that, the cell suspension was transferred to a pre-chilled tube with 0.5 mm silica beads and homogenized using a FastPrep^®^-24 (MP Biomedicals, SantaAna, CA, USA) (2 cycles of 30 s, 5 min resting on ice in between runs). The homogenized mixture was centrifuged at 7500 ×*g* for 30 min at 4 °C to remove insoluble cell debris. The cell-free extracts were stored at – 20 °C for further use.

### Phosphoketolase activity assay

Phosphoketolase activity was measured using the ferric hydroxamate method, based on the chemical conversion of enzymatically produced acetyl-phosphate into ferric acetyl hydroxamate, according to the protocol from [33]. The standard reactions were carried out in 1.5 mL of Eppendorf tube in a total assay volume of 100 μL consisting of 50 mM Tris (pH 7.5), 5 mM MgCl_2_, 5 mM potassium phosphate, 1 mM thiamine pyrophosphate and 10 mM F6P as a substrate. The crude cell-free extract was added to start the reaction and incubated at 30 °C for 1 h. To stop the enzymatic reaction, 60 μL hydroxylamine hydrochloride (2 M, pH 6.5) was added to 40 µL of assay solution. After incubation for 10 min at room temperature, 120 μL colouring reagent consisting of 15% (*w*/*v*) trichloroacetic acid, 4 M HCl, and FeCl_3_·6H_2_O (5% [*w*/*v*] in 0.1 M HCl) were added to generate ferric hydroxamate, which was then spectrophotometrically quantified at 505 nm by comparing to a series of lithium potassium acetyl-phosphate standards (Sigma).

### Analytical methods

Extracellular metabolite concentrations were determined by high-performance liquid chromatography (HPLC). Glycerol, xylose and mevalonate concentrations were detected and quantified using an ICS5000 HPLC (Thermo Scientific) with a refractive index detector (Shodex RI-101, sample frequency 5 Hz) and a Thermo UV/VIS detector (*λ* = 210 nm) coupled to an Animex HPX-87H column (BioRad) at 60 °C. Separations were performed using 0.016 N H_2_SO_4_ as an eluate at a flow rate of 0.6 mL/min. Culture samples were centrifuged at 16,000 ×g for 15 min. Supernatant and standards were mixed with 6 mM propionic acid as an internal standard at a ratio of 4:1. The flaviolin in the supernatant was quantified in triplicate in a Synergy microplate reader (Biotek Instruments) at a wavelength of 340 nm. The relative flaviolin production (N340) was determined by normalizing the measured absolute values (A340) by the cell density (OD_600_).

## Supplementary Information

**Additional file 1:** Raw data of growth and production experiments in the present study. 

**Additional file 2: Table S1.** Strains and plasmids used in the present study. **Table S2.** Primers used in this study. **Table S3.** Codon optimized xylose utilization genes and *xfpk* genes from different *Bifidobacterium* strains.

## Data Availability

All data generated and analysed in this study are included in this article and Additional files.
