# Supplementary material for: Increasing cellular fitness and product yields in Pseudomonas putida through an engineered phosphoketolase shunt
Source: Microb Cell Fact. 2023 Jan 19;22:14. doi: 10.1186/s12934-022-02015-9 (PMC9850600; doi:10.1186/s12934-022-02015-9)
Supplement: Supplementary file 2 — Additional file 2: Table S1. Strains and plasmids used in the present study. Table S2. Primers used in this study. Table S3. Codon optimized xylose utilization genes and xfpk genes from different Bifidobacterium strains. [file 12934_2022_2015_MOESM2_ESM.docx]

**Additional file data**

**Additional file 2: Table S1 Strains and plasmids used in the present study**

| **Strain or Plasmid** | **Characteristics** | **Source** |
| --- | --- | --- |
| ***Escherichia coli*** |  |  |
| Dh5α | Cloning host: F-λ-endA1 *glnX*44(AS) *thiE*1 *recA*1 *relA*1 *spoT*1 *gyrA*96(NalR) *rfbC*1 *deoR* *nupG* Φ80(*lacZ*ΔM15) Δ(*argF*-*lac*)U169 *hsdR*17(rK mK ) | Grant et al. (1990) |
| CC118λpir | Cloning host: araD139 Δ(*ara-leu*)7697 Δ*lacX*74 *galE* *galK* *phoA*20 *thi*− 1 *rpsE* *rpoB*(RifR) *argE*(Am) *recA*1, λpir lysogen | Herrero et al. (1990) |
| ***Pseudomonas putida*** |  |  |
| KT2440 | Wild-type strain; mt-2 derivative cured of the TOL plasmid pWW0 | Bagdasarian et al. (1982) |
| KT2440 Δ*glpR* | KT2440 with scarless deletion of *glpR* gene (PP_1074) | This work |
| KT2440 Δ*gcd* | KT2440 with scarless deletion of *gcd* gene (PP_1444) | This work |
| KT2440Δ*gcd*: XylABE | KT2440 Δ*gcd* with the *xylABE* operon integrated in the PP5322 locus | This work |
| KT2440Δ*gcd*: XylABE-Xfpk | KT2440 Δ*gcd* with the *xylABE-xfpk* operon integrated in the PP5322 locus | This work |
| **Plasmids** |  |  |
| pSEVAb83 | Expression vector: ori (pBBR1), Apr R | Damalas et al (2020) |
| pSEVAb62 | Expression vector: ori (RK2), Gm R | Damalas et al (2020) |
| pSEVAb23 | Expression vector: ori (pBBR1), Km R | Damalas et al (2020) |
| pGNW | Plasmid for genome editing in Gram-negative bacteria: ori (R6K) neo, KmR | Wirth et al. (2020) |
| pQURE6-H | Helper plasmid; oriV(RK2), *xylS*, Pm promoter, I-SceI; GmR | Volke et al., 2020 |
| pSEVA83b_*B.ani* | pSEVA83b containing the *xfpk* gene encoding xylulose 5-phosphate/fructose 6-phosphate phosphoketolase from *Bifidobacterium animalis* | This work |
| pSEVA83b_*B.ado* | pSEVA83b containing the *xfpk* gene encoding xylulose 5-phosphate/fructose 6-phosphate phosphoketolase from *Bifidobacterium adolescentis* | This work |
| pSEVA83b_*B.bre* | pSEVA83b containing the *xfpk* gene encoding xylulose 5-phosphate/fructose 6-phosphate phosphoketolase from *Bifidobacterium breve* | This work |
| pGNW-*glpR* | pGNW containing homology arms for scarless *glpR* deletion. | This work |
| pGNW-*gcd* | pGNW containing homology arms for scarless *gcd* deletion. | This work |
| pGNW - *XylABE* | pGNW containing the *xylABE* operon for integration | This work |
| pGNW – *Xyl-Xfpk* | pGNW containing the *xylABE-xfpk* operon for integration | This work |
| pSEVA62b-*B.bre* | pSEVA62b with *xfpk* gene encoding xylulose 5-phosphate/fructose 6-phosphate phosphoketolase from *Bifidobacterium breve* | This work |
| pSEVA23b - MAL | pSEVA23b with the *rppA* gene and the *accA-D* subunits from *P. putida* | This work |
| pSEVA23b - MVA | pSEVA23b containing *mvaE* and *mvaS* from *E. faecalis.* | This work |

**Additional file 2: Table S2 Primers used in this study.**

| **PCR Amplification primers** | | |
| --- | --- | --- |
| **Name** | **Sequence 5’ > 3’** | **Function** |
| LB_H1_glpR_FW | aggtctctcccgggcgtgaaggtagaccacatcatccg | Amplification of homology arm 1 for *glpR* deletion |
| LB_H1_glpR_RV | aggtctctgggcggtcctttggggctgc |  |
| LB_H2_glpR_FW | aggtctctgcccgggctggtgggtgcatgccg | Amplification of homology arm 2 for *glpR* deletion |
| LB_H2_glpR_RV | aggtctcttcgacacgatgtggggcgccttcgc |  |
| LB_H1_gcd_FW | aggtctctcccgcctaccgcagcagttcg | Amplification of homology arm 1 for *gcd* deletion |
| LB_H1_gcd_RV | aggtctctcgtaggttctccgtcaggtc |  |
| LB_H2_gcd_FW | aggtctcttacggcgacaccgctcccgcag | Amplification of homology arm 2 for *gcd* deletion |
| LB_H2_gcd_RV | aggtctcttcgactgccgaggtgtcgaagtggc |  |
| LB_Xpfk_B.bre_FW | attggtctcagaattcgcggccgcttctagatgacgattgattgggaacggga | Amplification of the Xfpk gene from Bifidobacterium breve. |
| LB_Xpfk_B.br_bsa1_RV | aggtctctataggtgccgtccaggtagg |  |
| LB_Xpfk_B.br_bsa1_FW | aggtctctctataccgaaaccttcccgaag |  |
| LB_Xpfk_B.br_bsa2_RV | aggtctctagcccggggtttccggagcg |  |
| LB_Xpfk_B.br_bsa2_FW | aggtctctggctccatccacgagggtg |  |
| LB_Xpfk_B.br_bsa3_RV | aggtctctttcgccgtcgccgacgatg |  |
| LB_Xpfk_B.br_bsa3_FW | aggtctctcgaagctgaaaccggcccgc |  |
| LB_Xpfk_B.br_bsa4_RV | aggtctctcagctcggcgaagcggc |  |
| LB_Xpfk_B.br_bsa4_FW | aggtctctgctgtgggaaaccatctgggac |  |
| LB_Xpfk_B.br_bsa5_RV | aggtctctggtccgaagatacggaagtcgc |  |
| LB_Xpfk_B.br_bsa5_FW | aggtctctgaccggatgaaaccgcttccaac |  |
| LB_Xpfk_B.br_bsa6_RV | aggtctctggttcatggaggcgatcggcttg |  |
| LB_Xpfk_B.br_bsa6_FW | aggtctctaacctgctcgtctcctcccacg |  |
| LB_Xpfk_B.bre_RV | taaggtctcgactgcagcggccgctactagtattattactcgttgtcgccagc |  |
| LB_Xfpk_B.ad_FW | cagtgctagctactagagaaagaggagaaatactagatgacgagtcctgttattggcacc | Amplification of the Xfpk gene from Bifidobacterium adolescentis. |
| LB_Xfpk_B.ad_RV | ggactgcagcggccgctactagtattattactcgttatcgccagcggttg |  |
| LB_Xfpk_B.an_FW | cagtgctagctactagagaaagaggagaaatactagatgactaatcctgttattggtac | Amplification of the Xfpk gene from Bifidobacterium animalis. |
| LB_Xfpk_B.an_RV | ggactgcagcggccgctactagtattattactcgttgtcgccggcgg |  |
| LB_MvaE_FW | attggtctcagaattcgcggccgcttctagatgaagacggtagttattatcg | Amplification of mvaE from E. facaelis |
| LB_MvaE_bsaI_RV | aggtctcttgatctgattttcaatcatatgg |  |
| LB_MvaE_bsaI_FW | aggtctctatcagcgaaaccgaggtgcc |  |
| LB_MvaE_RV | taaggtctcgactgcagcggccgctactagtattattactgtttgcgcaggtcattgagg |  |
| LB_MvaS_FW | attggtctcagaattcgcggccgcttctagatgaccattgggattgataaaatc | Amplification of mvaS from E. facaelis |
| LB_MvaS_RV | taaggtctcgactgcagcggccgctactagtattattaattgcgataggagcggacgg |  |

**Additional file 2: Table S3 Codon optimized xylose utilization genes and *xfpk* genes from different *Bifidobacterium* strains**

| ***XylA***  ATGCAGGCCTACTTCGACCAGCTGGACCGCGTGCGCTACGAAGGCTCGAAGTCGTCGAACCCGCTGGCCTTCCGCCACTACAACCCGGACGAACTGGTGCTGGGCAAGCGCATGGAAGAACACCTGCGCTTCGCCGCCTGCTACTGGCACACCTTCTGCTGGAACGGCGCCGACATGTTCGGCGTGGGCGCCTTCAACCGCCCGTGGCAGCAGCCGGGCGAAGCCCTGGCCCTGGCCAAGCGCAAGGCCGACGTGGCCTTCGAATTCTTCCACAAGCTGCACGTGCCGTTCTACTGCTTCCACGACGTGGACGTGTCGCCGGAAGGCGCCTCGCTGAAGGAATACATCAACAACTTCGCCCAGATGGTGGACGTGCTGGCCGGCAAGCAGGAAGAATCGGGCGTGAAGCTGCTGTGGGGCACCGCCAACTGCTTCACCAACCCGCGCTACGGCGCCGGCGCCGCCACCAACCCGGACCCGGAAGTGTTCTCGTGGGCCGCCACCCAGGTGGTGACCGCCATGGAAGCCACCCACAAGCTGGGCGGCGAAAACTACGTGCTGTGGGGCGGCCGCGAAGGCTACGAAACCCTGCTGAACACCGACCTGCGCCAGGAACGCGAACAGCTGGGCCGCTTCATGCAGATGGTGGTGGAACACAAGCACAAGATCGGCTTCCAGGGCACCCTGCTGATCGAACCGAAGCCGCAGGAACCGACCAAGCACCAGTACGACTACGACGCCGCCACCGTGTACGGCTTCCTGAAGCAGTTCGGCCTGGAAAAGGAAATCAAGCTGAACATCGAAGCCAACCACGCCACCCTGGCCGGCCACTCGTTCCACCACGAAATCGCCACCGCCATCGCCCTGGGCCTGTTCGGCTCGGTGGACGCCAACCGCGGCGACGCCCAGCTGGGCTGGGACACCGACCAGTTCCCGAACTCGGTGGAAGAAAACGCCCTGGTGATGTACGAAATCCTGAAGGCCGGCGGCTTCACCACCGGCGGCCTGAACTTCGACGCCAAGGTGCGCCGCCAGTCGACCGACAAGTACGACCTGTTCTACGGCCACATCGGCGCCATGGACACCATGGCCCTGGCCCTGAAGATCGCCGCCCGCATGATCGAAGACGGCGAACTGGACAAGCGCATCGCCCAGCGCTACTCGGGCTGGAACTCGGAACTGGGCCAGCAGATCCTGAAGGGCCAGATGTCGCTGGCCGACCTGGCCAAGTACGCCCAGGAACACCACCTGTCGCCGGTGCACCAGTCGGGCCGCCAGGAACAGCTGGAAAACCTGGTGAACCACTACCTGTTCGACAAGTAA |
| --- |
| ***XylB***  ATGTACATCGGCATCGACCTGGGCACCTCGGGCGTGAAGGTGATCCTGCTGAACGAACAGGGCGAAGTGGTGGCCGCCCAGACCGAAAAGCTGACCGTGTCGCGCCCGCACCCGCTGTGGTCGGAACAGGACCCGGAACAGTGGTGGCAGGCCACCGACCGCGCCATGAAGGCCCTGGGCGACCAGCACTCGCTGCAGGACGTGAAGGCCCTGGGCATCGCCGGCCAGATGCACGGCGCCACCCTGCTGGACGCCCAGCAGCGCGTGCTGCGCCCGGCCATCCTGTGGAACGACGGCCGCTGCGCCCAGGAATGCACCCTGCTGGAAGCCCGCGTGCCGCAGTCGCGCGTGATCACCGGCAACCTGATGATGCCGGGCTTCACCGCCCCGAAGCTGCTGTGGGTGCAGCGCCACGAACCGGAAATCTTCCGCCAGATCGACAAGGTGCTGCTGCCGAAGGACTACCTGCGCCTGCGCATGACCGGCGAATTCGCCTCGGACATGTCGGACGCCGCCGGCACCATGTGGCTGGACGTGGCCAAGCGCGACTGGTCGGACGTGATGCTGCAGGCCTGCGACCTGTCGCGCGACCAGATGCCGGCCCTGTACGAAGGCTCGGAAATCACCGGCGCCCTGCTGCCGGAAGTGGCCAAGGCCTGGGGCATGGCCACCGTGCCGGTGGTGGCCGGCGGCGGCGACAACGCCGCCGGCGCCGTGGGCGTGGGCATGGTGGACGCCAACCAGGCCATGCTGTCGCTGGGCACCTCGGGCGTGTACTTCGCCGTGTCGGAAGGCTTCCTGTCGAAGCCGGAATCGGCCGTGCACTCGTTCTGCCACGCCCTGCCGCAGCGCTGGCACCTGATGTCGGTGATGCTGTCGGCCGCCTCGTGCCTGGACTGGGCCGCCAAGCTGACCGGCCTGTCGAACGTGCCGGCCCTGATCGCCGCCGCCCAGCAGGCCGACGAATCGGCCGAACCGGTGTGGTTCCTGCCGTACCTGTCGGGCGAACGCACCCCGCACAACAACCCGCAGGCCAAGGGCGTGTTCTTCGGCCTGACCCACCAGCACGGCCCGAACGAACTGGCCCGCGCCGTGCTGGAAGGCGTGGGCTACGCCCTGGCCGACGGCATGGACGTGGTGCACGCCTGCGGCATCAAGCCGCAGTCGGTGACCCTGATCGGCGGCGGCGCCCGCTCGGAATACTGGCGCCAGATGCTGGCCGACATCTCGGGCCAGCAGCTGGACTACCGCACCGGCGGCGACGTGGGCCCGGCCCTGGGCGCCGCCCGCCTGGCCCAGATCGCCGCCAACCCGGAAAAGTCGCTGATCGAACTGCTGCCGCAGCTGCCGCTGGAACAGTCGCACCTGCCGGACGCCCAGCGCTACGCCGCCTACCAGCCGCGCCGCGAAACCTTCCGCCGCCTGTACCAGCAGCTGCTGCCGCTGATGGCCTAA |
| ***XylE***  ATGAACACCCAGTACAACTCGTCGTACATCTTCTCGATCACCCTGGTGGCCACCCTGGGCGGCCTGCTGTTCGGCTACGACACCGCCGTGATCTCGGGCACCGTGGAATCGCTGAACACCGTGTTCGTGGCCCCGCAGAACCTGTCGGAATCGGCCGCCAACTCGCTGCTGGGCTTCTGCGTGGCCTCGGCCCTGATCGGCTGCATCATCGGCGGCGCCCTGGGCGGCTACTGCTCGAACCGCTTCGGCCGCCGCGACTCGCTGAAGATCGCCGCCGTGCTGTTCTTCATCTCGGGCGTGGGCTCGGCCTGGCCGGAACTGGGCTTCACCTCGATCAACCCGGACAACACCGTGCCGGTGTACCTGGCCGGCTACGTGCCGGAATTCGTGATCTACCGCATCATCGGCGGCATCGGCGTGGGCCTGGCCTCGATGCTGTCGCCGATGTACATCGCCGAACTGGCCCCGGCCCACATCCGCGGCAAGCTGGTGTCGTTCAACCAGTTCGCCATCATCTTCGGCCAGCTGCTGGTGTACTGCGTGAACTACTTCATCGCCCGCTCGGGCGACGCCTCGTGGCTGAACACCGACGGCTGGCGCTACATGTTCGCCTCGGAATGCATCCCGGCCCTGCTGTTCCTGATGCTGCTGTACACCGTGCCGGAATCGCCGCGCTGGCTGATGTCGCGCGGCAAGCAGGAACAGGCCGAAGGCATCCTGCGCAAGATCATGGGCAACACCCTGGCCACCCAGGCCGTGCAGGAAATCAAGCACTCGCTGGACCACGGCCGCAAGACCGGCGGCCGCCTGCTGATGTTCGGCGTGGGCGTGATCGTGATCGGCGTGATGCTGTCGATCTTCCAGCAGTTCGTGGGCATCAACGTGGTGCTGTACTACGCCCCGGAAGTGTTCAAGACCCTGGGCGCCTCGACCGACATCGCCCTGCTGCAGACCATCATCGTGGGCGTGATCAACCTGACCTTCACCGTGCTGGCCATCATGACCGTGGACAAGTTCGGCCGCAAGCCGCTGCAGATCATCGGCGCCCTGGGCATGGCCATCGGCATGTTCTCGCTGGGCACCGCCTTCTACACCCAGGCCCCGGGCATCGTGGCCCTGCTGTCGATGCTGTTCTACGTGGCCGCCTTCGCCATGTCGTGGGGCCCGGTGTGCTGGGTGCTGCTGTCGGAAATCTTCCCGAACGCCATCCGCGGCAAGGCCCTGGCCATCGCCGTGGCCGCCCAGTGGCTGGCCAACTACTTCGTGTCGTGGACCTTCCCGATGATGGACAAGAACTCGTGGCTGGTGGCCCACTTCCACAACGGCTTCTCGTACTGGATCTACGGCTGCATGGGCGTGCTGGCCGCCCTGTTCATGTGGAAGTTCGTGCCGGAAACCAAGGGCAAGACCCTGGAAGAACTGGAAGCCCTGTGGGAACCGGAAACCAAGAAGACCCAGCAGACCGCCACCCTGTAA |
| ***xfpk* from *B. breve***  **ATGACGATTGATTGGGAACGGGAATCCTTGGCGCCGCAAGGAACCAAGGCCCGCGACCTAACGGAAATAATCAATCGCACGATCACGTGCAGGAGTACAGGAGTACACATGACGAGTCCTGTTATTGGCACCCCTTGGAAGAAGCTCAACGCTCCGGTTTCCGAGGAGTCCCTCGAAGGCGTTGACAAGTACTGGCGCGTTGCCAACTACCTTTCCATCGGCCAGATTTATCTGCGTTCCAACCCGCTGATGAAGGCTCCCTTCACCCGCGAAGATGTGAAGCACCGTCTGGTCGGCCACTGGGGCACTACCCCTGGCCTGAACTTCCTCATCGGCCACATCAACCGCTTCATCGCTGACCACGGCCAGAACACCGTGATCATCATGGGCCCGGGCCACGGTGGCCCGGCTGGTACCTCCCAGTCCTACCTGGACGGCACCTATACCGAGACCTTCCCGAAGATTACCAAGGATGAGGCTGGTCTGCAGAAGTTCTTCCGTCAGTTCTCCTACCCGGGCGGTATCCCGTCCCACTTCGCTCCGGAGACCCCGGGCTCCATCCACGAGGGTGGTGAGCTGGGCTACGCTCTGTCTCACGCTTACGGCGCCATCATGGACAACCCGAGCCTGTTCGTCCCGGCCATCGTCGGCGACGGCGAAGCTGAGACCGGCCCGCTGGCTACCGGCTGGCAGTCCAACAAGCTCGTGAACCCGCGCACCGACGGTATCGTGCTGCCGATCCTGCACCTCAACGGCTACAAGATCGCCAACCCGACCATCCTGTCCCGCATCTCCGACGAAGAGCTCCACGAGTTCTTCCACGGCATGGGTTACGAGCCCTACGAGTTCGTCGCTGGCTTCGACGATGAAGATCACATGTCCATCCACCGCCGCTTCGCCGAGCTGTGGGAGACCATCTGGGACGAGATCTGCGACATCAAGGCCGCCGCTCAGACCGACAACGTGCACCGTCCGTTCTACCCGATGCTGATCTTCCGCACCCCGAAGGGTTGGACCTGCCCGAAGTACATCGACGGCAAGAAGACCGAGGGCTCCTGGCGCGCTCACCAGGTGCCGTTGGCTTCCGCCCGCGACACCGAGGCCCACTTCGAGGTCCTCAAGAACTGGCTCGAGTCCTACAAGCCGGAAGAGCTGTTCGACGCCAACGGCGCTGTCAAGGACGACGTCCTTGCCTTCATGCCGAAGGGTGAGCTGCGTATCGGTGCCAACCCGAACGCCAACGGTGGTGTGATTCGCGACGACCTGAAGCTGCCGAACCTCGAGGACTACGAGGTCAAGGAAGTGGCTGAGTACGGCCACGGCTGGGGCCAGCTCGAGGCCACCCGTACCCTGGGTGCCTACACTCGTGACATCATCCGCAACAACCCGCGCGACTTCCGTATCTTCGGACCGGATGAGACCGCTTCCAACCGTCTGCAGGCTTCCTACGAGGTCACCAACAAGCAGTGGGATGCTGGCTACATCTCCGATGAGGTCGACGAGCACATGCACGTCTCCGGCCAGGTCGTCGAGCAGCTGTCCGAGCACCAGATGGAAGGCTTCCTCGAGGCCTACCTGCTGACCGGTCGTCACGGTATCTGGAGCTCCTACGAGTCCTTCGTCCACGTGATCGACTCCATGCTCAACCAGCACGCCAAGTGGCTTGAGGCTACCGTCCGCGAGATTCCGTGGCGCAAGCCGATCGCCTCCATGAACCTGCTGGTCTCCTCCCACGTTTGGCGTCAGGACCACAACGGCTTCTCCCACCAGGACCCGGGTGTCACCTCCGTCCTGCTGAACAAGTGCTTCCACAACGACCACGTCATCGGCATCTACTTCGCCACCGATGCGAACATGCTGCTGGCCATCGCCGAGAAGTGCTACAAGTCCACCAACAAGATCAACGCCATCATCGCCGGCAAGCAGCCCGCTGCCACCTGGCTGACCCTGGACGAGGCTCGTGCCGAGCTCGCCAAGGGTGCCGCCGCTTGGGATTGGGCTTCCACCGCCAAGAACAACGATGAGGCCGAGGTCGTGCTCGCCGCCGCTGGCGACGTCCCGACCCAGGAGATCATGGCTGCTTCTGACAAGCTGAAGGAACTGGGCGTCAAGTTCAAGGTTGTGAACGTTGCCGACCTGCTCTCTCTGCAGTCTGCCAAGGAGAACGACGAGGCTCTGAGCGACGAGGAGTTCGCTGACATCTTCACCGCCGACAAGCCGGTGCTGTTCGCATACCACTCCTACGCTCACGACGTGCGCGGTCTGATCTACGATCGTCCGAACCACGACAACTTCAACGTCCACGGCTACGAGGAGGAGGGCTCCACCACCACCCCGTACGACATGGTTCGCGTCAACCGCATCGACCGCTACGAGCTGACCGCCGAGACTCTGCGCATGATCGACGCCGACAAGTACGCCGACAAGATCGACGAGCTCGAGAAGTTCCGTGACGAGGCCTTCCAGTTCGCCGTCGACAAGGGCTACGACCACCCGGACTACACCGACTGGGTGTACTCCGGCGTGAACACCGACAAGAAGGGCGCCGTCACCGCCACTGCCGCCACCGCTGGCGACAACGAGTGA** |
| ***xfpk* from *B. adolescentis***  **ATGACGAGTCCTGTTATTGGCACCCCTTGGAAGAAGCTGAACGCTCCGGTTTCCGAGGAAGCTATCGAAGGCGTGGATAAGTACTGGCGCGCAGCCAACTACCTCTCCATCGGCCAGATCTATCTGCGTAGCAACCCGCTGATGAAGGAGCCTTTCACCCGCGAAGACGTCAAGCACCGTCTGGTCGGTCACTGGGGCACCACCCCGGGCCTGAACTTCCTCATCGGCCACATCAACCGTCTCATTGCTGATCACCAGCAGAACACTGTGATCATCATGGGCCCGGGCCACGGCGGCCCGGCTGGTACCGCTCAGTCCTACCTGGACGGCACCTACACCGAGTACTTCCCGAACATCACCAAGGATGAGGCTGGCCTGCAGAAGTTCTTCCGCCAGTTCTCCTACCCGGGTGGCATCCCGTCCCACTACGCTCCGGAGACCCCGGGCTCCATCCACGAAGGCGGCGAGCTGGGTTACGCCCTGTCCCACGCCTACGGCGCTGTGATGAACAACCCGAGCCTGTTCGTCCCGGCCATCGTCGGCGACGGTGAAGCTGAGACCGGCCCGCTGGCCACCGGCTGGCAGTCCAACAAGCTCATCAACCCGCGCACCGACGGTATCGTGCTGCCGATCCTGCACCTCAACGGCTACAAGATCGCCAACCCGACCATCCTGTCCCGCATCTCCGACGAAGAGCTCCACGAGTTCTTCCACGGCATGGGCTATGAGCCGTACGAGTTCGTCGCTGGCTTCGACAACGAGGATCACCTGTCGATCCACCGTCGTTTCGCCGAGCTGTTCGAGACCGTCTTCGACGAGATCTGCGACATCAAGGCCGCCGCTCAGACCGACGACATGACTCGTCCGTTCTACCCGATGATCATCTTCCGTACCCCGAAGGGCTGGACCTGCCCGAAGTTCATCGACGGCAAGAAGACCGAGGGCTCCTGGCGTTCCCACCAGGTGCCGCTGGCTTCCGCCCGCGATACCGAGGCCCACTTCGAGGTCCTCAAGAACTGGCTCGAGTCCTACAAGCCGGAAGAGCTGTTCGACGAGAACGGCGCCGTGAAGCCGGAAGTCACCGCCTTCATGCCGACCGGCGAACTGCGCATCGGTGAGAACCCGAACGCCAACGGTGGCCGCATCCGCGAAGAGCTGAAGCTGCCGAAGCTGGAAGACTACGAGGTCAAGGAAGTCGCCGAGTACGGCCACGGCTGGGGCCAGCTCGAGGCCACCCGTCGTCTGGGCGTCTACACCCGCGACATCATCAAGAACAACCCGGACTCCTTCCGTATCTTCGGACCGGATGAGACCGCTTCCAACCGTCTGCAGGCCGCTTACGACGTCACCAACAAGCAGTGGGACGCCGGCTACCTGTCCGCTCAGGTCGACGAGCACATGGCTGTCACCGGCCAGGTCACCGAGCAGCTTTCCGAGCACCAGATGGAAGGCTTCCTCGAGGGCTACCTGCTGACCGGCCGTCACGGCATCTGGAGCTCCTATGAGTCCTTCGTGCACGTGATCGACTCCATGCTGAACCAGCACGCCAAGTGGCTCGAGGCTACCGTCCGCGAGATTCCGTGGCGCAAGCCGATCTCCTCCATGAACCTGCTCGTCTCCTCCCACGTGTGGCGTCAGGATCACAACGGCTTCTCCCACCAGGATCCGGGTGTCACCTCCGTCCTGCTGAACAAGTGCTTCAACAACGATCACGTGATCGGCATCTACTTCCCGGTGGATTCCAACATGCTGCTCGCTGTGGCTGAGAAGTGCTACAAGTCCACCAACAAGATCAACGCCATCATCGCCGGCAAGCAGCCGGCCGCCACCTGGCTGACCCTGGACGAAGCTCGCGCCGAGCTCGAGAAGGGTGCTGCCGAGTGGAAGTGGGCTTCCAACGTGAAGTCCAACGATGAGGCTCAGATCGTGCTCGCCGCCACCGGTGATGTTCCGACTCAGGAAATCATGGCCGCTGCCGACAAGCTGGGCGCCATGGGCATCAAGTTCAAGGTCGTCAACGTGGTTGACCTGGTCAAGCTGCAGTCCGCCAAGGAGAACAACGAGGCCCTCTCCGATGAGGAGTTCGCTGAGCTGTTCACCGAGGACAAGCCGGTCCTGTTCGCTTACCACTCCTATGCCCGCGATGTGCGTGGTCTGATCTACGATCGCCCGAACCACGACAACTTCAACGTTCACGGCTACGAGGAGCAGGGCTCCACCACCACCCCGTACGACATGGTTCGCGTGAACAACATCGATCGCTACGAGCTCCAGGCTGAAGCTCTGCGCATGATTGACGCTGACAAGTACGCCGACAAGATCAACGAGCTCGAGGCCTTCCGTCAGGAAGCCTTCCAGTTCGCTGTCGACAACGGCTACGATCACCCGGATTACACCGACTGGGTCTACTCCGGTGTCAACACCAACAAGCAGGGTGCTATCTCCGCTACCGCCGCAACCGCTGGCGACAACGAGTGA** |
| ***xfpk* from *B. animalis***  **ATGACTAATCCTGTTATTGGTACCCCATGGCAGAAGCTGGATCGTCCGGTTTCCGAAGAGGCCATCGAAGGCATGGACAAGTACTGGCGCGTCGCCAACTACATGTCTATCGGCCAGATCTACCTGCGTAGCAACCCGCTGATGAAGGAGCCCTTCACCCGCGATGACGTGAAGCACCGTCTGGTCGGCCACTGGGGCACCACCCCGGGCCTGAACTTCCTTCTCGCCCACATCAACCGCCTGATCGCCGATCACCAGCAGAACACCGTGTTCATCATGGGTCCTGGCCACGGCGGCCCTGCAGGTACCGCTCAGTCCTACATCGACGGCACCTACACCGAGTACTACCCGAACATCACCAAGGACGAAGCTGGCCTGCAGAAGTTCTTCCGCCAGTTCTCCTACCCGGGTGGCATTCCTTCCCACTTCGCTCCGGAGACGCCGGGCTCCATCCACGAAGGCGGCGAGCTGGGCTACGCCCTGTCGCACGCCTACGGCGCGATCATGGACAACCCGAGCCTCTTCGTCCCGTGCATCATCGGTGACGGCGAAGCCGAGACCGGCCCTCTGGCCACCGGCTGGCAGTCCAACAAGCTCGTCAACCCGCGCACCGACGGCATCGTCCTGCCGATCCTGCACCTCAACGGCTACAAGATCGCCAACCCGACGATCCTCGCCCGCATCTCCGACGAGGAGCTGCACGACTTCTTCCGCGGCATGGGTTACCACCCGTACGAGTTCGTCGCCGGCTTCGACAACGAGGATCACCTGTCGATCCACCGTCGCTTCGCCGAGCTCTTCGAGACCATCTTCGACGAGATCTGCGATATCAAGGCTGCGGCTCAGACCGACGACATGACCCGTCCGTTCTACCCGATGCTCATCTTCCGCACCCCGAAGGGCTGGACCTGCCCGAAGTTCATCGACGGCAAGAAGACCGAAGGCTCCTGGCGTGCACACCAGGTCCCGCTGGCTTCCGCCCGCGACACCGAGGCCCACTTCGAAGTCCTCAAGGGCTGGATGGAATCCTACAAGCCGGAGGAGCTCTTCAACGCCGACGGCTCCATCAAGGAGGACGTCACCGCATTCATGCCTAAGGGCGAACTGCGCATCGGCGCCAACCCGAATGCCAACGGCGGCCGCATCCGCGAGGATCTGAAGCTCCCTGAGCTCGATCAGTACGAGATCACCGGCGTCAAGGAATACGGCCACGGTTGGGGCCAGGTCGAGGCTCCGCGTTCCCTCGGCGCGTACTGCCGCGACATCATCAAGAACAACCCGGATTCGTTCCGCGTCTTCGGACCTGACGAGACCGCGTCCAACCGTCTGAACGCGACCTACGAGGTCACCAAGAAGCAGTGGGACAACGGATACCTCTCGGCTCTCGTCGACGAGAACATGGCCGTCACCGGCCAGGTTGTCGAGCAGCTCTCCGAGCATCAGTGCGAAGGCTTCCTCGAGGCCTACCTGCTCACCGGCCGTCACGGCATCTGGAGCTCCTACGAGTCCTTCGTGCACGTGATCGACTCCATGCTGAACCAGCATGCGAAGTGGCTCGAGGCCACCGTCCGCGAGATCCCGTGGCGTAAGCCGATCTCCTCGGTGAACCTCCTGGTCTCCTCGCACGTGTGGCGTCAGGATCACAACGGCTTCTCGCACCAGGATCCGGGTGTGACCTCCGTCCTGCTGAACAAGACGTTCAACAACGACCACGTGACGAACATCTACTTCGCGACCGATGCCAACATGCTGCTGGCCATCGCCGAGAAGTGCTTCAAGTCCACCAACAAGATCAACGCAATCTTCGCCGGCAAGCAGCCGGCCGCGACGTGGATCACCCTCGACGAGGTACGCGCCGAGCTCGAGGCTGGTGCCGCCGAGTGGAAGTGGGCTTCCAACGCCAAGAGCAACGACGAGGTCCAGGTTGTCCTCGCCGCCGCCGGCGACGTCCCGACCCAGGAGATCATGGCCGCTTCCGATGCCCTCAACAAGATGGGCATCAAGTTCAAGGTCGTCAACGTCGTGGACCTCATCAAGCTGCAGTCCTCGAAGGAGAACGACGAGGCCATGTCTGACGAGGACTTCGCCGACCTGTTCACCGCGGACAAGCCGGTCCTCTTCGCCTACCACTCCTATGCCCAGGACGTTCGTGGCCTCATCTACGACCGCCCGAACCACGACAACTTCACCGTTGTCGGATACAAGGAGCAGGGCTCCACGACGACGCCGTTCGACATGGTGCGTGTCAACGACATGGATCGCTACGCCCTTCAGGCCAAGGCCCTCGAGCTCATCGACGCCGACAAGTATGCCGACAAGATCAACGAGCTCAACGAGTTCCGCAAGACCGCGTTCCAGTTCGCCGTCGACAATGGCTATGACATTCCTGAGTTCACCGATTGGGTGTACCCGGATGTCAAGGTCGACGAGACCTCCATGCTCTCCGCCACCGCCGCGACCGCCGGCGACAACGAGTAA** |
